# The Hokkaido Birth Cohort Study on Environment and Children’s Health: cohort profile—updated 2017

**DOI:** 10.1186/s12199-017-0654-3

**Published:** 2017-05-18

**Authors:** Reiko Kishi, Atsuko Araki, Machiko Minatoya, Tomoyuki Hanaoka, Chihiro Miyashita, Sachiko Itoh, Sumitaka Kobayashi, Yu Ait Bamai, Keiko Yamazaki, Ryu Miura, Naomi Tamura, Kumiko Ito, Houman Goudarzi, the members of The Hokkaido Study on Environment and Children’s Health, M. Hoshiba, N. Kanda, M. Kihara, R. Nakanishi, A. Kondo, H. Sato

**Affiliations:** 1 Hokkaido University Center for Environmental and Health Sciences, Kita 12, Nishi 7, Kita-ku, Sapporo, 060-0812 Japan; 2 Graduate School of Health Sciences, Hokkaido University, Sapporo, Japan; 3 Department of Public Health, Graduate School of Medicine, Hokkaido University, Sapporo, Japan; 4 Department of Respiratory Medicine, Faculty of Medicine and Graduate School of Medicine, Hokkaido University, Sapporo, Japan

**Keywords:** Birth cohort study, Environmental chemicals, Exposure measurement, Pregnancy outcomes, Birth size, Thyroid, reproductive, and steroid hormones, Neurobehavioral development, Allergies and infectious diseases, Genetic susceptibility, Epigenetics

## Abstract

The Hokkaido Study on Environment and Children’s Health is an ongoing study consisting of two birth cohorts of different population sizes: the Sapporo cohort and the Hokkaido cohort. Our primary study goals are (1) to examine the effects of low-level environmental chemical exposures on birth outcomes, including birth defects and growth retardation; (2) to follow the development of allergies, infectious diseases, and neurobehavioral developmental disorders and perform a longitudinal observation of child development; (3) to identify high-risk groups based on genetic susceptibility to environmental chemicals; and (4) to identify the additive effects of various chemicals, including tobacco smoking. The purpose of this report is to update the progress of the Hokkaido Study, to summarize the recent results, and to suggest future directions. In particular, this report provides the basic characteristics of the cohort populations, discusses the population remaining in the cohorts and those who were lost to follow-up at birth, and introduces the newly added follow-up studies and case-cohort study design. In the Sapporo cohort of 514 enrolled pregnant women, various specimens, including maternal and cord blood, maternal hair, and breast milk, were collected for the assessment of exposures to dioxins, polychlorinated biphenyls, organochlorine pesticides, perfluoroalkyl substances, phthalates, bisphenol A, and methylmercury. As follow-ups, face-to-face neurobehavioral developmental tests were conducted at several different ages. In the Hokkaido cohort of 20,926 enrolled pregnant women, the prevalence of complicated pregnancies and birth outcomes, such as miscarriage, stillbirth, low birth weight, preterm birth, and small for gestational age were examined. The levels of exposure to environmental chemicals were relatively low in these study populations compared to those reported previously. We also studied environmental chemical exposure in association with health outcomes, including birth size, neonatal hormone levels, neurobehavioral development, asthma, allergies, and infectious diseases. In addition, genetic and epigenetic analyses were conducted. The results of this study demonstrate the effects of environmental chemical exposures on genetically susceptible populations and on DNA methylation. Further study and continuous follow-up are necessary to elucidate the combined effects of chemical exposure on health outcomes.

## Background

In 1997, Colborn et al. warned of the dangers of environmental chemicals as endocrine disruptors, which could lead to impaired reproductive capacity [1]. Since that warning, a myriad of animal and epidemiological studies have evaluated the adverse health effects of these endocrine-disrupting chemicals (EDCs) [2–4]. Although the use of polychlorinated dibenzo-*p*-dioxins (PCDDs), polychlorinated dibenzofurans (PCDFs), polychlorinated biphenyls (PCBs), perfluorooctanoic sulfonate (PFOS), and perfluorooctanoic acid (PFOA) is regulated, these chemicals are still detected in the environment and in the human body. Concentrations of PFOS and PFOA, the conventional perfluoroalkyl substances (PFASs), have been decreasing; however, the concentrations of newly developed PFASs such as perfluorononanoic acid (PFNA) and perfluorodecanoic acid (PFDA) have been increasing [5]. Accumulating evidence has shown the various adverse health effects of exposure to environmental chemicals; however, few studies have focused on vulnerable populations such as fetuses and children. Thus, together with the Developmental Origins of Health and Disease (DOHaD) theory, which proposes that the early life environment has widespread consequences for later health, this prospective birth cohort study was established.

The Hokkaido Study on Environment and Children’s Health: Malformation, Development and Allergy is an ongoing birth cohort study launched in 2002. The Hokkaido Study is one of the oldest birth cohort studies not only in Japan but also in Asia [6]. The Hokkaido Study consists of two cohorts of different population sizes; the Sapporo cohort mainly focuses on the deep investigation and understanding of child neurobehavioral development by taking advantage of the relatively small number of participants and study area, while the Hokkaido cohort focuses on rare diseases such as birth defects and developmental disorders (e.g., autism spectrum disorder and attention deficit hyperactivity disorder) which are not commonly found in small cohorts. These two cohorts of different sizes allow us to examine various outcomes including child growth, development, allergy, and infectious diseases.

Our primary study goals include (1) examining the possible negative effects of perinatal environmental chemical exposure on birth outcomes, including birth defects and growth retardation; (2) following the development of allergies, infectious diseases, and neurobehavioral developmental disorders and performing a longitudinal observation of child development; (3) identifying high-risk groups with genetic susceptibilities to environmental chemical exposures; and (4) identifying the additive effects of lifestyle including tobacco smoking and folic acid intake encountered in the daily environment.

Our previous cohort profile update in 2013 [7] introduced our findings since the beginning of the study. We reported that higher concentrations of toxic equivalents (TEQ) of dioxin and other specific congeners in the maternal blood increased the adverse effects on infant birth weight, neurobehavioral development, and immune function. However, we were only able to produce limited reports on the effects of gene-environment interactions between environmental chemicals and maternal/infant genes on adverse health outcomes.

Over the 14 years of follow-ups, new research questions have arisen and the study focus has expanded beyond its primary goals. Studies have reported an increasing prevalence of children with developmental disabilities and behavioral problems [8, 9]. The prevalence of childhood allergic conditions is also increasing [10, 11]. These childhood health issues are of global public health concern; thus, the longitudinal observations of our cohort study will provide evidence on the associations between the in utero environment and child health outcomes.

Emerging evidence has indicated that epigenetic modifications including DNA methylation may link the intrauterine environment to lifelong health trajectories [12]. The effects of in utero environmental chemical exposure on DNA methylation in the developing fetus are gaining increasing attention. The wide range of data collected in this cohort study allows us to further investigate new research questions including DNA methylation and its health outcomes.

The purpose of this report is to update the progress of the Hokkaido Study, to summarize the recent results, and to suggest its future directions. In particular, this report focuses on (1) providing the basic characteristics of the whole cohort population as the fixed data are now available after the closing of recruitment in 2012, and presenting the comparisons of the population remaining in the cohort with those who were lost to follow-up at birth; (2) introducing the nested case-cohort study design, in which a sub-cohort is used as a comparison group for all cases that occur in the cohort; and (3) introducing the details of the follow-up studies, including child neurobehavioral problems at preschool ages. This design is effective when a cohort is followed for health outcomes by avoiding the costs of collecting and processing covariate information and exposure assessment in the selected sub-cohort population. This strategy is applied to the Hokkaido cohort, which contains more than 20,000 participants.

## Methods

### Study areas, participants, and baseline questionnaire

The details of the Hokkaido Study have been described in previous reports [7, 13]. Briefly, the Hokkaido Study consists of two birth cohorts: the Sapporo cohort and the Hokkaido (large-scale) cohort. Two cohorts differed in terms of the strategies. The Sapporo cohort enrolled 514 pregnant women at 23–35 weeks of gestation who planned to deliver at Toho Hospital in Sapporo city from July 2002 to October 2005. Among 1796 potentially eligible women, the women had incomplete partner’s information, the women had decided to enroll in the Japanese cord blood bank (22% of those of approached), or the women decided to deliver the baby at another hospital (3% of those of approached) was excluded. Ultimately, 514 pregnant women agreed to participate. The Hokkaido cohort enrolled pregnant women before 13 weeks of gestational age who visited one of the associated hospitals or clinics of three university hospitals in the Hokkaido prefecture from February 2003 to March 2012. The participation rate was 55%. Thirty-seven associated hospitals or clinics, including three university hospitals, were evenly distributed throughout the Hokkaido prefecture, accounting for approximately 40% of the institutes with delivery units in this prefecture [14]. Parent and infant medical records were obtained from physicians at the participating hospitals or clinics. Both the Sapporo and Hokkaido cohorts were conducted after obtaining written informed consent from all participants. The institutional ethical board for human gene and genome studies at Hokkaido University Center for Environmental and Health Sciences and Hokkaido University Graduate School of Medicine approved the study protocol.

We also established a sub-cohort of 4869 participants, which corresponded to 23.3% of all participants in the Hokkaido Study. In this sub-cohort, we included 500 participants who were randomly selected from each enrollment year between 2003 and 2011, and all 369 participants from the enrollment year 2012.

### Follow-up and outcome measures: the Sapporo cohort

Details of the information collected at the time of enrollment and birth have been described previously [7, 13]. A self-administered questionnaire was completed at the time of enrollment to obtain parental baseline information. Maternal medical and infant birth records were obtained from the hospital. Follow-up studies were conducted at 6 and 18 months and 3.5 and 7 years of age, which collected information regarding childhood neurological and behavioral development and medical histories of asthma, allergies, and infectious diseases. Measurements of the second and fourth digits were made from photocopies of the ventral surface of the right and left hands at school age; the ratio of the second finger to fourth finger lengths (2D/4D) was calculated in order to evaluate the effects of prenatal Leydig cell function [15].

The details of the follow-up questionnaires and tests are shown in Table 1.

**Table 1:** Follow-ups performed at different ages

	Neurobehavioral development	Allergy	Anthropometric measurements/puberty
Sapporo cohort			
6–7 months	BSID-II, FTII, EES		
1.5 years	BSID-II, DDST, EES	ISAAC	Health checkup data
3.5 years	K-ABC, CBCL, WAIS-R, EES	ISAAC	Health checkup data
7 years	WISC-III, WCST-KFS, CBCL, J-PSAI, 2D/4D	ISAAC	Health checkup data
12 years			Tanner staging, onset of puberty
Hokkaido cohort			
1 year		ISAAC, ATS-DLD	Health checkup data
1.5 years	M-CHAT		
2 years		ISAAC	Health checkup data
3 years	KIDS, SDQ		
4 years		ISAAC	Health checkup data
5 years	SDQ, DCDQ		
6 years	ADHD-RS, ASQ		
7 years		ISAAC, home visit	Health checkup data
8 years	ADHD-RS, Conners 3P, ASQ, CBCL, J-PSAI, WISC-IV		
9 years			Tanner staging, onset of puberty

*ADHD-RS* Attention Deficit Hyperactivity Disorder-Rating Scale, *ASQ* Autism Screening Questionnaire, *ATS-DLD* American Thoracic Society-Division of Lung Disease, *M-CHAT* Modified Checklist for Autism in Toddlers, *BSID-II* Bayley Scales of Infant Development second edition, *CBCL* Child Behavior Checklist, *Conners 3P* Conner’s 3rd Edition for Parents, *DCDQ* Developmental Coordination Disorder Questionnaire, *DDST* Denver Developmental Screening Tests, *EES* Evaluation of Environmental Stimulation, *FTII* Fagan Test of Infant Intelligence, *ISAAC* International Study of Asthma and Allergies in Childhood, *J-PSAI* Japanese Pre-School Activities Inventory, *K-ABC* Kaufman Assessment Battery for Children, *KIDS* Kinder Infant Development Scale, *SCQ* Social Communication Questionnaire, SDQ Strengths and Difficulties Questionnaire, *WAIS-R* Wechsler Adult Intelligence Scale-Revised, *WISC-III* Wechsler Intelligence Scale for Children third edition, *WCST-KFS* Wisconsin Card Sorting Test (Keio Version), *WISC-IV* Wechsler Intelligence Scale for Children fourth edition

### Follow-up and outcome measures: the Hokkaido cohort

Observations were made to assess the prevalence of birth defects, asthma and allergies, and neurobehavioral developmental disorders [7]. The definitions of birth outcomes were as follows: Very low birth weight (VLBW) was birth weight <1500 g, extremely low birth weight (ELBW) was birth weight <1000 g, and small for gestational age (SGA) was defined as birth weights lower than the 10th percentile of the normative reference birth weight, per gestational age, sex, and parity, among live-born infants. Additionally, new follow-ups particularly focusing on the socioeconomic status (SES) and child developmental and behavioral problems have begun since the last cohort profile update. In these follow-ups, we also obtained information on childcare environment and maternal mental health and stress. The 1.5- and 3-year-old follow-ups started in October 2013 and January 2015, using widely used questionnaires, namely the Modified Checklist for Autism in Toddlers (M-CHAT) at 1.5 years old and Kinder Infant Development Scale (KIDS) and Strengths and Difficulties Questionnaire (SDQ) at 3 years old, respectively. The follow-ups of 5- and 6-year-old participants started in October 2014. Table 1 contains a detailed description of the follow-up questionnaires and tests.

### Specimen collection and measurements

The details of specimen collection are described in our previous cohort profile [7]. In the Sapporo cohort, maternal non-fasting blood samples were collected at the time of the hospital examination following recruitment, at the 23rd–35th week of gestation. Samples of cord blood, placenta, maternal hair, and breast milk from nursing mothers were collected after delivery. In the Hokkaido cohort, maternal non-fasting blood samples were collected during the first and the third trimester and at delivery. Cord blood samples were collected immediately after the birth. Table 2 summarizes the samples measured for exposures and biochemical markers along with the measurement methods in the Hokkaido Study.

**Table 2:** Items measured in The Hokkaido Study on Environment and Children’s Health

Measurement	Method	Specimen
Exposure measurements		
Dioxins, PCBs, OH-PCBs (congener level)	HRGC/HRMS	Maternal blood, milk
PFOS, PFOA, and other PFASs	UPLC-MS/MS	Maternal blood
Phthalate metabolites	GC-MS, LC-MS/MS	Maternal blood, child urine
Chlorinated pesticides	GC-NCIMS, GC-HRMS	Maternal blood
Cotinine	ELISA	Maternal and cord blood
Bisphenol A	ID-LC-MS/MS	Maternal and cord blood
Me-Hg	Oxygen combustion-gold amalgamation method	Maternal hair
Phthalate esters and organophosphate flame retardants	GC-MS	House dust
Mite allergens	ELISA	House dust
Biological measurements		
Thyroid hormones (TSH, FT3, FT)	ELISA	Maternal and cord blood
Folic acid	CLEIA	Maternal blood
Fatty acids (palmitic, stearic, palmitoleic, oleic, linoleic, arachidonic, α-linolenic, EPA, DHA)	GC-MS	Maternal blood
TG	TC E-Test Wako	Maternal blood
IgE, IgA	ELISA	Cord blood
Adipokines (adiponectin, leptin)	ELISA and RIA	Cord blood
Steroid hormones (estradiol, testosterone, progesterone, cortisol, cortisone, DHEA, androstenedione)	LC-MS/MS	Cord blood
Reproductive hormones (inhibin B, INSL-3, SHBG, FSH, LH)	ELISA, RIA, EIA	Cord blood

*DHA* docosahexaenoic acid, *DHEA* dehydroepiandrosterone, *EPA* eicosapentaenoic acid, *FSH* follicle-stimulating hormone, *FT4* free thyroxine, *IgA* immunoglobulin A, *IgE* immunoglobulin E, *INSL-3* insulin-like factor 3, *LH* luteinizing hormone, *Me-Hg* methylmercury, *OH*-*PCB* hydroxylated polychlorinated biphenyl, *PCB* polychlorinated biphenyl, *PFASs* perfluorinated alkyl substances, *PFOA* perfluorooctanoic acid, *PFOS* perfluorooctanoic sulfonate, *SHBG* sex hormone-binding globulin, *TG* triglyceride, *TSH* thyroid-stimulating hormone

In the Sapporo cohort, the levels of 29 dioxin and dioxin-like PCB (DL-PCB) congeners, 58 other PCB congeners, and 5 hydroxylated PCB (OH-PCB) congeners in maternal blood and breast milk were measured [16–18]. The PFOS and PFOA levels in maternal blood, cord blood, and breast milk were analyzed at Hoshi University [19]. The levels of 29 persistent organochlorine pesticides, mono(2-ethylhexyl) phthalate (MEHP) (a primary metabolite of di(2-ethylhexyl) phthalate (DEHP)), and bisphenol A in maternal blood were measured. Bisphenol A levels in cord blood were also analyzed. Finally, total mercury (Hg) levels in maternal hair samples were measured [20].

In the Hokkaido cohort, simultaneous analysis of 11 PFASs in maternal plasma collected during the third trimester of pregnancy was conducted [5]. In addition, cotinine concentrations in maternal plasma were measured. Based on self-reports and plasma cotinine levels using receiver operating characteristic curve analysis, cutoff values of 0.21 and 11.48 ng/mL were established to separate smokers and exposed nonsmokers from unexposed nonsmokers, respectively [21]. Household dust and child urine samples were collected at 7 years [7].

As outcomes, thyroid, steroid, and reproductive hormone levels were measured using cord blood samples. Adipokine levels were also measured in cord blood samples. In addition to hormone levels, IgE and IgA levels were measured.

### Genetic and epigenetic analysis

Genotyping of single nucleotide polymorphisms (SNPs) was performed using allelic discrimination assays, with fluorogenic probes and 5′ nuclease (TaqMan) (Applied Biosystems, Foster, CA, USA) [22] or a nanofluidic integrated fluidic circuit-based genotyping system for the medium-throughput multiplexing of 96 individual human DNA samples and 96 individual SNP assays (Dynamic Array, Fluidigm Corporation, South San Francisco, CA, USA) [23–25]. In the Sapporo cohort, offspring patterns of insulin-like growth factor 2 (*IGF2*), *H19*, and long interspersed element 1 (*LINE1*) DNA methylation in cord blood were analyzed by pyrosequencing using a PyroMark Q24 system (Qiagen, Hilden, Germany) and assessed using the Qiagen Pyro Q-CpG Software [26]. Cord blood DNA methylation at more than 450,000 CpG sites was quantified using an Infinium HumanMethylation450 BeadChip (Illumina, San Diego, CA, USA) according to the manufacturer’s protocol [27, 28].

## Results

### Participant characteristics and birth outcomes

The fixed dataset as of the end of 2015 included 20,926 women who had initially registered. However, 121 of the participants requested that their data not be included in any analysis; thus, we included data from 20,805 participants. The basic characteristics of the Sapporo (*n* = 514) and Hokkaido (*n* = 20,805) cohorts are shown in Tables 3 and 4, respectively. In the Sapporo cohort, 10 participants dropped out before delivery. There are 7 pairs of twins in the Sapporo cohort. Among 497 singleton neonates, the prevalence of preterm delivery, low birth weight, and small for gestational age (SGA) was 5.6, 5.8, and 6.2%, respectively. As shown in Table 4, the follow-up rate of the Hokkaido cohort is 94.1%. In the Hokkaido cohort, we defined those whose data was not available in the medical records at delivery as lost to follow-ups. The participants who were lost to follow-up showed younger maternal age, higher prevalence of nulliparity and drinking, and lower prevalence of passive smoking. The proportions of missing values in the questionnaire were comparable between the two groups (Table 4). Characteristics of participants in follow-ups and in sub-cohort were comparable. We considered sub-cohort population reasonably represented whole cohort population, and thus, the sub-cohort populations can be used for future nested case-cohort study.

**Table 3:** Basic characteristics of the Sapporo cohort participants (*n* = 514)

	Number	Mean (±SD), Med (25–75%), or %
Maternal characteristics		
Age at entry (years)	510	30.4 ± 4.9
Maternal body mass index	506	21.2 ± 3.2
Nulliparous (%)	240	47.7
Education (years)		
≤9	14	2.8
10–12	211	41.5
13–16	274	53.9
≥17	9	1.8
Household income (million yen/year)		
<3	95	18.8
3 to <5	250	49.5
5 to <7	105	20.8
7 to 10	48	9.5
≥10	7	1.4
Drinking habit during pregnancy (%)	157	30.9
Smoking habit during pregnancy (%)	103	20.3
Caffeine intake during pregnancy (mg/day)	508	124 (76–188)
Paternal characteristics		
Age at entry (years)	503	32.3 ± 5.6
Education (years)		
≤9	31	6.1
10–12	190	37.5
13–16	242	47.8
≥17	43	8.5
Smoking habit during pregnancy (%)	354	69.8
Child characteristics		
Sex, boys (%)	246	48.1
Gestational age (weeks)	504	38.9 ± 1.5
Birth weight (g)	511	3025.6 ± 420.7
Birth length (cm)	511	47.9 ± 2.2
Types of delivery, vaginal (%)	397	78.8

Missing data were excluded from the calculation

*SGA* small for gestational age

**Table 4:** Comparison of the characteristics of the Hokkaido cohort participants included and lost to follow-up

	Follow-ups (*n* = 19,579)			Lost to follow-up (*n* = 1226)			Sub-cohort (*n* = 4869)		
	*n*	Mean or %	95% CI	*n*	Mean or %	95% CI	*n*	Mean or %	95% CI
Maternal characteristics									
Age at entry (years)	19,549	29.9	29.8–29.9	1166	29.4	29.1–29.7	4831	29.9	29.8–30.0
Maternal body mass index	18,428	21.2	21.1–21.2	1141	20.9	20.7–21.0	4608	21.2	21.1–21.3
Nulliparous (%)	7226	42.1	41.4–42.9	595	54.5	51.5–57.4	1726	40.9	39.4–42.3
History of reproductive therapy (%)	925	4.9	4.6–5.3	73	6.2	5.0–7.8	259	5.5	4.9–6.2
Regular use of any medicine (%)	1956	10.3	9.8–10.7	139	11.3	9.7–13.2	519	11.0	10.1–11.9
Regular use of any supplement (%)	5852	30.7	30.0–31.3	378	30.8	28.3–33.5	1565	33.1	31.8–34.5
Drinking habit at 1st trimester (%)	2180	11.7	11.3–12.2	141	12.1	10.4–14.1	531	11.4	10.5–12.4
Smoking habit 1st trimester (%)	2136	12.0	11.6–12.5	135	12.2	10.5–14.3	487	11.6	10.7–12.6
Passive smoking in house (%)	11,752	65.9	65.2–66.6	687	61.2	58.3–64.0	2852	63.6	62.2–65.0
Education, compulsory education only (%)	1011	5.4	5.1–5.7	74	6.4	5.1–7.9	247	5.3	4.7–6.0
Occupation, no job	8137	41.6	40.9–42.3	499	40.7	38.0–43.5	2012	41.5	40.1–42.9
Income, <3,000,000 yen/year (%)	3662	22.8	22.2–23.5	240	23.8	21.2–26.5	916	22.7	21.4–24.0
Paternal characteristics									
Age at entry (years)	18,311	31.7	31.6–31.8	1135	31.1	30.8–31.4	4595	31.7	31.5–31.9
Drinking habit (%)	13,375	72.1	71.5–72.8	863	75.2	72.6–77.6	3319	71.5	70.2–72.8
Smoking habit 1st trimester (%)	10,855	66.4	65.7–67.1	650	64.3	61.3–67.2	2644	65.0	63.5–66.4
Education, compulsory education only (%)	1431	7.8	7.4–8.2	78	6.9	5.6–8.5	347	7.5	6.8–8.3
Occupation, no job	417	2.2	2.0–2.4	41	3.4	2.6–4.7	117	2.5	2.0–3.0

“Lost to follow-ups” included participants who dropped out before delivery or whose data at birth had not been recorded. Missing data were excluded from the calculation

*CI* confidence interval

The prevalence of pregnancy complications was also examined. The most common complication was pregnancy hypertension, observed in 491 women (2.5%). Other complications included gestational diabetes mellitus (159, 0.8%), pregnancy with preexisting diabetes (34, 0.2%), oligoamnios (205, 1.1%), thyroid dysfunction (133, 0.7%), placenta previa (18, 0.1%), and placental abruption (16, 0.1%). Table 5 shows the outcomes of singleton births. The incidences of spontaneous abortion, stillbirths, and preterm birth of singleton fetuses were 1.1, 0.32, and 4.9%, respectively. We observed low birth weight (LBW) and macrosomia in 9.0 and 1.0% of births, respectively. The incidences of VLBW and ELBW among singleton deliveries were 1.4 and 1.1%, respectively. Based on the Japan Pediatric Society database of birth weights [29], the incidences of SGA and term SGA in this study were 7.0 and 6.5%, respectively. All birth defects after 12 weeks of gestation, including 55 marker anomalies, were recorded. The prevalence of all birth defects was 18.9/1000 births. Approximately 10% of all patients with birth defects were delivered between 12 and 21 weeks of gestation. Among births with congenital malformations of the nervous system, 39% were delivered before 22 weeks of gestation. All patients with anencephaly and encephalocele were delivered before 22 weeks of gestation [30].

**Table 5:** Outcomes of singleton fetuses (*n* = 18,882) at birth in the Hokkaido cohort

Outcomes	Number	Percent	95% CI
Miscarriage^a^	200	1.1	0.9–1.2
Artificial abortion^b^	52	0.28	0.21–0.36
Stillbirth^c^	60	0.32	0.24–0.41
Live birth	18,570	98.3	98.2–98.5
Preterm birth^d^	923	4.9	4.6–5.2
Moderate preterm birth	795	4.2	3.9–4.5
Very preterm birth	112	0.59	0.49–0.71
Extremely preterm birth	47	0.24	0.18–0.33
Low birth weight^e^	1693	9.0	8.6–9.4
Very low birth weight	268	1.4	1.2–1.6
Extremely low birth weight	204	1.1	0.9–1.2
Macrosomia^f^	190	1.0	0.9–1.2
Small for gestational age^g^	1308	7.0	6.7–7.4
Term small for gestational age^h^	1211	6.5	6.2–6.9
Small for reference fetal weight^i^	814	4.8	4.5–5.2

Missing data were excluded from the calculation

*CI* confidence interval

^a^Miscarriage was defined as the loss of a pregnancy at <22 completed gestational weeks

^b^An abortion brought about intentionally at <22 completed gestational weeks

^c^Stillbirth was defined as the birth of a dead fetus at ≥22 completed gestational weeks

^d^Preterm birth was defined as birth at 22–36 completed gestational weeks. Preterm birth was subdivided into three categories of prematurity: moderately preterm (32–36 completed weeks), very preterm (28–31 completed weeks), and extremely preterm (22–27 completed weeks)

^e^Low birth weight, very low birth weight, and extremely low birth weight were birth weights <2500, <1500, and <1000 g, respectively

^f^Macrosomia is birth weight ≥4000 g

^g^Small for gestational age infants had birth weights less than the 10th percentile of the reference birth weight estimated by using gestational age, sex, and parity

^h^Term small for gestational age infants were the small for gestational age infants among the term birth neonates

^i^Small for reference fetal weight infants had birth weights <1.5 standard deviations of the reference ultrasound-based fetal weight estimated from gestational age, sex, and parity

### Exposure levels of environmental chemicals

The exposure levels of environmental chemicals in the Sapporo cohort are shown in Table 6. The median levels of total TEQ and total PCBs in maternal blood were 13.9 pg/g lipid and 107 ng/g lipid, respectively. The median levels of PFOS and PFOA were 5.20 and 1.30 ng/mL, respectively. Among 29 organochlorine pesticides, the highest concentration detected was 651.99 pg/g wet mass of *p*,*p′*-dichlorodiphenyldichloroethylene (*p*,*p′*-DDE). The detailed concentrations of organochlorine pesticides can be found in our previous literature [31]. MEHP was detected in all 493 maternal blood samples, with a median concentration of 9.95 ng/mL. Concentrations (detection percentages above the detection limit) of bisphenol A in maternal and cord blood were 0.057 ng/mL (68.8%) and 0.051 ng/mL (76.3%), respectively. Finally, total Hg concentration in maternal hair was 1.40 μg/g.

**Table 6:** Exposure levels of environmental chemicals in the Sapporo cohort

					Percentile			
	Number	DL	>DL (%)	Min	25th	50th	75th	Max
Maternal blood								
Total dioxins (TEQ pg/g lipid)	426	n/a	n/a	3.17	9.95	13.9	18.2	43.4
Total PCBs (ng/g lipid)	426	n/a	n/a	17.8	73.0	107	148	41,460
*p*,*p′*-DDE	379	0.60	100	99.52	401.53	650.99	1011.48	4575.67
PFOS (ng/mL)	447	0.5	100	1.30	3.40	5.20	7.00	16.2
PFOA (ng/mL)	447	0.5	92.8	0.25	0.80	1.30	1.80	5.30
MEHP (ng/mL)	493	0.278	100	1.94	5.82	9.95	16.3	101.7
Bisphenol A (ng/mL)	59	0.04	76.3	<DL	0.040	0.057	0.072	0.419
Cord blood								
Bisphenol A (ng/mL)	285	0.04	68.8	<DL	<DL	0.051	0.076	0.217
Maternal hair								
Me-Hg (μg/g)	430	n/a	100	0.24	0.96	1.40	1.89	7.55

*p,p′-DDE* *p,p′*-dichlorodiphenyldichloroethylene, *DL* detection limit, *Me-Hg* methylmercury, *MEHP* mono(2-ethylhexyl) phthalate, *n/a* not applicable, *PCBs* polychlorinated biphenyls, *PFOA* perfluorooctanoic acid, *PFOS* perfluorooctanoic sulfonate, *TEQ* toxicity equivalency quantity

In the Hokkaido cohort, the concentrations of 11 PFASs in the third trimester maternal plasma were measured (Table 6). PFOS and PFOA were detected in all samples, and the detection percentages of PFNA, PFDA, perfluorodecanoic acid, perfluorotridecanoic acid (PFTrDA), perfluorododecanoic acid (PFDoDA), and perfluorohexane sulfonate (PFHxS) were above 80%, whereas the detection percentages of perfluorohexanoic acid, perfluoroheptanoic acid, and perfluorotetradecanoic acid were less than 50% [32]. The measurements of dioxins, PCBs, phthalates, and BPA are currently ongoing in the Hokkaido cohort.

### Health effects

#### Birth size

The effects of PCDD/PCDF and DL-PCB, PFOS, and PFOA exposure on birth size were presented in our 2012 cohort profile [33, 34]. Since the last cohort profile update [7], prenatal exposure to other chemicals including PCBs, total Hg, and DEHP have been investigated in association with birth size. The results are shown in Table 7. We observed that PCDD/PCDFs and PFASs were associated with decreased birth weight [33, 34]; however, DEHP, PCBs, and total Hg were not associated with birth weight [35, 36]. Our investigation not only was on conventional birth weight but also included cord adipokines considered to be biomarkers of metabolic function [35, 37].

**Table 7:** Findings from the Sapporo cohort on the associations between exposures and birth size

Exposures	Outcome	Number	Findings	Reference
PCDD/PCDFs	Birth weight	398	Significant decrease. Individual congener assessment found 2,3,4,7,8-PeCDF had a significant negative influence (per log10 unit: *β* = *−*24.5 g, 95% CI *−*387.4 to *−*61.5).	[33]
PCBs	Birth weight	367	No association.	[36]
PFASs	Birth weight	428	PFOS was negatively correlated (per log10 unit: *β* = *−*269.4 g, 95% CI *−*465.7 to *−*73.0).	[34]
	Ponderal index	177	PFOA was negatively associated (per log10 unit: *β* = *−*0.73 kg/m^3^, 95% CI *−*1.44 to *−*0.02).	[26]
	Cord adipokines	168	PFOS was positively associated with adiponectin (per log10 unit: *β* = 0.12, 95% CI 0.01 to 0.22).	[37]
DEHP	Ponderal index	167	Significant decrease (per log10 unit: *β* = −1.28, 95% CI *−*2.43 to *−*0.13).	[35]
	Cord adipokines	167	Significant decrease in leptin among girls (per log10 unit: *β* = −0.31, 95% CI *−*0.52 to *−*0.10). Significant increase in adiponectin among boys (per log10 unit: *β* = 4.63, 95% CI 0.77 to 8.49).	
Me-Hg	Birth weight/SGA	367	No association with birth weight. The risk of SGA by weight decreased with increasing Me-Hg.	[36]

*PCDD* polychlorinated dibenzo-*p*-dioxin, *PCDF* polychlorinated dibenzofuran, *PCB* polychlorinated biphenyls, *PFASs* perfluorinated alkyl substances, *PFOS* perfluorooctanoic sulfonate, *PFOA* perfluorooctanoic acid, *DEHP* di(2-ethylhexyl) phthalate, *Me-Hg* methylmercury, *SGA* small for gestational age

#### Thyroid, reproductive, and steroid hormone levels

Maternal and infant thyroid hormone levels were investigated in association with prenatal chemical exposure [38–41]. The findings suggested that PFOS possibly disrupted both maternal and infant thyroid hormone levels and that Σdioxin levels were possibly associated with increased infant free thyroxin levels (Table 8).

**Table 8:** Findings from the Sapporo cohort on the association between exposures and hormone levels at birth

Exposures	Outcome	Number	Findings	Reference
PFAS	Maternal/infant TSH, FT4	392	Maternal PFOS levels were inversely correlated with maternal serum TSH and positively associated with infant serum TSH levels, whereas maternal PFOA showed no significant relationship with TSH or FT4 levels among mothers and infants.	[38]
DEHP	Infant TSH, FT4	328	No association.	[39]
Bisphenol A	Infant TSH, FT4	285	No association.	[40]
PCB	Infant TSH, FT4	358	Log10 Σdioxin (TEQ) was associated with increased FT4 (*β* = 0.224 ng/dL, 95% CI 0.016 to 0.433) overall, and the association was more significant among boys (*β* = 0.299 ng/dL, 95% CI 0.011 to 0.587).	[41]
PFASs	Cord reproductive hormones	189	PFOS levels were positively associated with E2 and T/E2 and inversely with P4; inhibin B and PFOA levels were positively associated with inhibin B levels in boys. Significant inverse associations were observed between PFOS levels and P4 and PRL levels in girls.	[45]
	Cord steroid hormones	185	Cortisol and cortisone reduced in association with PFOS level (Q4 vs. Q1: *β* = *−*23.98 ng/mL, 95% CI *−*47.12 to *−*11.99; *β* = *−*63.21 ng/mL, 95% CI *−*132.56 to *−*26.72). DEHA increased (Q4 vs. Q1: *β* = 1.33 ng/mL, 95% CI 0.17 to 1.82). DHEA decreased (Q4 vs. Q1: *β* = *−*1.23 ng/mL, 95% CI *−*1.72 to *−*0.25) in association with PFOA.	[44]
DEHP	Cord reproductive hormones	202	MEHP was associated with reduced levels of T/E2, P4, and inhibin B. Inverse associations between maternal MEHP levels T/E2, P4, inhibin B, and INSL3 for males.	[42]
	Cord steroid hormones	202	MEHP was associated with reduced cortisol and cortisone levels and glucocorticoid/adrenal androgen ratio whereas increased DHEA levels and DHEA/androstenedione ratio.	[43]
Bisphenol A	Cord reproductive hormones	285	Negatively associated with PRL (*β* = *−*0.38).	[40]

*DEHP* di(2-ethylhexyl) phthalate, *DHEA* dehydroepiandrostenedione, *E2* estradiol, *INSL3* insulin-like factor 3, *Me-Hg* methylmercury, *MEHP* mono(2-ethylhexyl) phthalate, *P4* progesterone, *PCDD* polychlorinated dibenzo-*p*-dioxin, *PCDF* polychlorinated dibenzofuran, *PCB* polychlorinated biphenyls, *PFASs* perfluorinated alkyl substances, *PFOS* perfluorooctanoic sulfonate, *PFOA* perfluorooctanoic acid, *PRL* prolactin, *T* testosterone, *TSH* thyroid-stimulating hormone, *FT4* free thyroxine

Steroid and reproductive hormone levels were also investigated in the Sapporo cohort (Table 8). We examined the relationship between prenatal exposure to PFOS, PFOA, DEHP, and bisphenol A and the levels of steroid and reproductive hormones in cord blood [40, 42–45]. Prenatal exposure to PFOS, but not PFOA, showed an inverse dose-response relationship with cortisol and cortisone concentrations. The DHEA level was positively associated with PFOS concentration and negatively associated with PFOA concentration. We observed no significant associations between PFASs and androstenedione levels. PFOS was associated with decreased cortisol/DHEA and glucocorticoid/androgenic hormone ratios, indicating that PFOS may shift steroidogenesis to androgenic hormones [44]. PFOS and PFOA showed both positive and inverse associations with the levels of several reproductive and steroid hormones [44, 45]. Similarly, MEHP was associated with reduced levels of several reproductive and steroid hormones, especially in boys [42, 43]. Bisphenol A was associated with reduced prolactin levels [40]. Some of the associations observed in this study between environmental chemicals, including PFASs, DEHP, and bisphenol A, and cord hormone levels were sex-specific.

#### Neurobehavioral development

In addition to the effects of PCDD/PCDF on the Bayley Scales of Infant Development second edition scores at 6 months reported in our previous update [7, 46], the associations between PFASs, DEHP, and bisphenol A exposure and childhood neurobehavioral development were investigated using the same assessment tool [39, 47, 48]. The results are shown in Table 9. PFOA, but not PFOS, adversely affected mental development in girls at 6 months of age [47]. DEHP and bisphenol A exposures were not associated with child mental or psychomotor development [39, 40]. Stronger adverse effects of prenatal exposure to dioxins on neurodevelopmental have recently been reported in male children [49]. Prenatal environments such as SES are reportedly associated with child behavioral problems [50] and intellectual ability [51].

**Table 9:** Findings from the Sapporo and Hokkaido cohorts on the relationship between exposures and neurodevelopment

Exposures	Outcome	Age at testing	Number	Findings	Reference
Sapporo cohort					
PCDD/PCDFs	BSID-II	6 months	134	Several dioxin isomers showed adverse effects on motor development in 6-month-old male infants.	[46]
PCDD/PCDFs, PCBs	BSID-II	18 months	190	At 18 months of age, the associations observed at 6 months disappeared. The levels of six dioxin isomers were significantly positively associated with mental development in 18-month-old girls.	[49]
PFASs	BSID-II	6 and 18 months	173	PFOA was negatively associated with mental development in 6-month-old girls (per log10 unit: *β* = *−*0.296, 95% CI *−*11.96 to *−*0.682).	[47]
DEHP	BSID-II	6 and 18 months	328	Not associated.	[39]
Bisphenol A	BSID-II, CBCL, K-ABC	6 and 18 months 3.5 years 3.5 years	285	Not associated with mental and psychomotor development, but associated with internalizing problems at 42 months (per log10 unit: *β* = 4.37, 95% CI 0.11 to 8.64).	[48]
SES	K-ABC	3.5 years	145	Family income is an optimum indicator of SES in the association with intellectual ability in Japanese children aged 42 months.	[51]
Hokkaido cohort					
SES	SDQ	5 years	2553	Maternal prepregnancy BMI ≥30 kg/m^2^, primipara, maternal education ≤high school, family income during pregnancy <3 million yen/year, and boy gender were the factors associated with increased odds ratio of the likelihood of child behavioral problems.	[50]

*BSID-II* Bayley Scales of Infant Development second edition, *CBCL* Child Behavior Checklist, *DEHP* di(2-ethylhexyl) phthalate, *K-ABC* Kaufman Assessment Battery for Children, *Me-Hg* methylmercury, *PCB* polychlorinated biphenyls, *PCDD* polychlorinated dibenzo-*p*-dioxin, *PCDF* polychlorinated dibenzofuran, *PFASs* perfluorinated alkyl substances, *PFOS* perfluorooctanoic sulfonate, *PFOA* perfluorooctanoic acid, *SDQ* Strengths and Difficulties Questionnaire, *SES* socioeconomic status, *BMI* body mass index

#### Allergies and infectious diseases

The prevalence rates of wheezing, eczema, food allergy, and otitis media at 1.5 years of age in the Sapporo cohort were 11.3, 11.3, 17.0, and 18.7%, respectively. In the Hokkaido cohort, the prevalence rates of wheezing, eczema, and allergic rhino-conjunctivitis symptoms at 2 years of age were 19.3, 17.8, and 4.4%, respectively, and at 4 years of age were 18.7, 19.0, and 5.4%, respectively. The prevalence rates observed in the Hokkaido Study are slightly higher than those reported in the Japanese population [52].

Since our last cohort update [7, 53, 54], the associations between prenatal exposure to PFASs, including long-chain compounds, and allergic diseases at 1, 2, and 4 years of age have been investigated. The main findings are shown in Table 10. Overall, newly emerged PFASs such as PFDoDA and PFTrDA were associated with reduced infant allergies, whereas PFOS increased the risk of infectious diseases [32, 55, 56].

**Table 10:** Findings from the Sapporo and Hokkaido cohorts on the relationships between exposures and allergies and infectious diseases

Exposures	Outcome	Number	Findings	Reference
Sapporo cohort				
Dioxins	Otitis media	364	Polychlorinated dibenzofuran was associated with increased risk among male infants (OR 2.5, 95% CI 1.1–5.9). 2,3,4,7,8-pentachlorodibenzo-furan was associated with increased risk of otitis media (OR 5.3, 95% CI 1.5–19).	[53]
PFASs	Cord IgE/infectious disease	343	Cord IgE levels decreased with high maternal PFOA concentration among females. No associations among maternal PFOS and PFOA levels and food allergy, eczema, wheezing, or otitis media in the 18-month-old infants.	[54]
Hokkaido cohort				
PFASs	Eczema	2063	At 24 months, the risk in association with higher maternal PFTrDA levels decreased (OR 0.62, 95% CI 0.45–0.86).	[32]
	Total allergic diseases/eczema/wheezing	1558	ORs in the Q4 vs. Q1 for total allergic diseases decreased for PFDoDA (OR 0.621, 95% CI 0.454–0.847) and PFTrDA (OR 0.712, 95% CI 0.524–0.966). OR (Q4 vs. Q1) for wheezing in relation to higher maternal PFHxS levels was 0.728 (95% CI 0.497–1.06).	[56]
	Infectious diseases	1558	PFHxS was associated with higher risk of total infections disease among girls (Q1 vs. Q4: OR 1.55, 95% CI 0.976–2.45).	[55]

*IgE* immunoglobulin E, *Q* quartile, *OR* odds ratio, *PFASs* perfluorinated alkyl substances, *PFDoDA* perfluorododecanoic acid, *PFHxS* perfluorohexane sulfonate, *PFOA* perfluorooctanoic acid, *PFOS* perfluorooctanoic sulfonate, *PFTrDA* perfluorotridecanoic acid, *CI* confidence interval

#### Maternal fatty acid concentrations during pregnancy

Maternal fatty acids (FAs) are essential for fetal growth. In our study, nine types of FAs and triglycerides (TGs) were investigated in association with PFAS and DEHP exposure. Our study found that PFOS but not PFOA was negatively associated with the levels of palmitic, palmitoleic, oleic, linoleic, α-linolenic, and arachidonic acids and TG. PFOS was associated with reduced essential FAs, including omega-3 and omega-6 FAs [57]. We also found that a tenfold increase in blood MEHP levels was correlated with a decrease in TG levels as well as similar relationships in palmitic, oleic, linoleic, and α-linolenic acids [58]. These were the first studies to address the association between PFASs, DEHP, and FAs in pregnant women. The results provided important evidence regarding the association of relatively low levels of PFOS or DEHP with the concentrations of essential and long-chain polyunsaturated FAs.

#### Effects of exposure and gene polymorphisms

The effects of maternal smoking during pregnancy and maternal genetic polymorphisms on infant birth weight were examined in the Sapporo and Hokkaido cohorts [22, 59–63]. The results are shown in Table 11. In both cohorts, birth weight was significantly lower among infants born to smoking mothers having certain genetic variations, e.g., in the genes aromatic hydrocarbon receptor (*AhR*), cytochrome P450 1A1 (*CYP1A1*), glutathione *S*-transferase mu 1, nicotinamide adenine dinucleotide phosphate, cytochrome P450 2EI, 5,10-methylenetetrahydrofolate reductase, and X-ray cross-complementing gene 1.

**Table 11:** Adverse effects of infant birth weight in relation to maternal environmental exposure and genetic polymorphisms

Environmental exposure	Maternal genetic polymorphism	Maternal risk genotypes	Birth weight reduction (g)	Reference
Active smoking	*AhR* (G>A, Arg554Lys)	Arg/Arg	*−*211	[22]
	*CYP1A1* (m1/m2)	m1/m2 + m2/m2	*−*170	
	*GSTM1* (non-null/null)	Null	*−*171	
	Combination with *AhR* (G>A, Arg554Lys) and *CYP1A1* (m1/m2)	Arg/Arg (*AhR*) and m1/m2 + m2/m2 (*CYP1A1*)	*−*315	
	Combination with *CYP1A1* (m1/m2) and *GSTM1* (non-null/null)	m1/m2 + m2/m2 (*CYP1A1*) and null (*GSTM1*)	*−*237	
	*NQO1* (C>T, Pro187Ser)	Pro/Pro	*−*159	[62]
	*CYP2E1* (c1/c2)	c1/c1	*−*195	
	*MTHFR* (A1298C)	AA	*−*106	[63]
	*CYP1A1* (A>G, Ile462Val)	AG/GG	*−*62	[61]
	*XRCC1* (C>T, Arg194Trp)	CT/TT	*−*59	
	Combination with *AhR* (G>A, Arg554Lys), *CYP1A1* (A>G, Ile462Val), and *XRCC1* (C>T, Arg194Trp)	GG (*AhR*), AG/GG (*CYP1A1*), and CT/TT (*XRCC1*)	*−*145	
Dioxin (total TEQ)	*GSTM1* (non-null/null)	Null	*−*345	[59]

*AhR*, aromatic hydrocarbon receptor, *CYP1A1* cytochrome P450 1A1, *CYP2E1* cytochrome P450 2E1, *GSTM1* glutathione *S*-transferase mu 1, *MTHFR*, methylenetetrahydrofolate reductase, *NQO1* nicotinamide adenine dinucleotide phosphate quinone oxidase 1, *XRCC1* X-ray cross-complementing gene 1, *TEQ* toxicity equivalency quantity

**Fig. 1:**
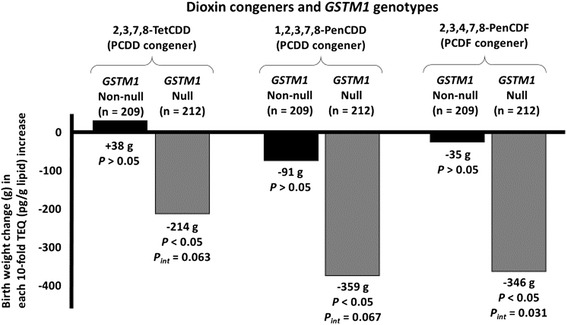
Association of maternal PCDD and PCDF congeners and maternal GSTM1 (non-null/null) genotypes with reduction of infant birth weight in the multiple linear regression model (*N* = 421) [59]

The associations between maternal exposure to dioxins during pregnancy and maternal genetic polymorphisms were also examined in the Sapporo cohort. Maternal polymorphisms in the *AhR* (G>A, Arg554Lys) and *CYP1A1* (T>C, MspI) genes were associated with maternal dioxin concentration [60]. Moreover, among 29 dioxin congeners, reduced infant birth weight was associated with increased levels of certain dioxin congeners [59] (Fig. 1).

#### Effects of epigenetic modification

In multiple linear regression models, *IGF2* methylation was significantly inversely associated with log-unit increases in PFOA levels but had no significant effects on *H19* or *LINE1* methylation after full adjustment [26]. *IGF2* methylation was significantly positively associated with ponderal index values at birth but was not associated with birth weight or birth length. Mediation analysis [64] showed a significant indirect effect of PFOA exposure on ponderal index via *IGF2* methylation. Remarkably, around one fifth of the effects of prenatal PFOA exposure on reduced ponderal index can be explained by methylation at only the *IGF2* gene. These results indicated that the effects of prenatal PFOA exposure could be mediated through DNA methylation. Further study is required to determine the potential for long-term adverse health effects of DNA methylation changes induced by PFOA exposure.

## Discussion

### Exposure levels

The mean total dioxin TEQ level of maternal blood in this study was 13.9 TEQ pg/g lipid, which was lower than the levels reported in other studies in Taiwan, Europe, the USA, and even in Japan [33]. Concentrations of PCB 153 in maternal serum have been reported in several different birth cohorts, with median concentrations ranging from 10.7 ng/g lipid weight in Poland to 450 ng/g lipid in the Faroe Islands [36]. In the present study, the maternal PCB 153 concentration of 21.0 ng/g was considerably lower than that among populations in Europe and similar to that of the eastern coast of the USA, South Korea, and Taiwan [36].

Similar to those of dioxins and PCBs, the levels of PFOS and PFOA measured in the Sapporo cohort were lower than those reported in studies in Europe, the USA, and other Asian countries [65–70]. In the Hokkaido cohort, a temporal trend analysis of PFAS levels from 2003 to 2011 revealed that PFOS and PFOA concentrations declined, whereas PFNA and PFDA concentrations increased [5]. There are limited data regarding long-chain PFAS concentrations in different populations worldwide. Thus, it is important to evaluate the long-term trends in the levels of these compounds in humans. Among non-DL-PCBs, PCDDs/PCDFs and DL-PCBs, PFOS, PFOA, and methylmercury (Me-Hg), almost all chemicals were significantly correlated [20]. *p*,*p*′-DDE exposure levels in this study were comparable to those of two previous studies in Japan, indicating that exposure levels are low in Japan [31].

The levels of MEHP detected in this study were slightly higher than those in American adults, elderly Swedish, and pregnant Australian women, although the exposure levels were not exactly comparable to those of the other studies due to the differences in measurement methods [42]. The slightly higher MEHP levels in this study can be acceptable as the levels of DEHP in household dust in Sapporo were higher than those found in studies from other countries, and DEHP intake in the Sapporo population was higher than that of most other studies [71, 72]. The relatively lower detection limit in this study made it possible to evaluate the lower levels of total bisphenol A observed in the Sapporo cohort. Although the correlation was not significant, bisphenol A levels in maternal and cord blood were similar; thus, the placenta may not reduce or protect the transfer of bisphenol A from the mother to the fetus [73].

### Birth outcomes

In Japan, spontaneous abortion is defined as the death of the fetus between 12 and 21 weeks of gestational age [74]. The proportion of stillbirths among all births was 3.3 per 1000 in the Hokkaido area in 2012 [75]. We observed a comparable proportion (3.2 per 1000) in this study. The proportion of preterm births among singleton deliveries was 4.8% in 2012 according to the National Vital Statistics of Japan [75], comparable to the proportion (4.9%) observed in the present study.

Birth weight is a commonly used variable in perinatal research. In the Hokkaido area, the mean birth weight in 2012 was 3010 g [75]. According to the National Vital Statistics, the average birth weight has recently decreased [74]. Increased mortality has been historically observed in neonates with low weight. Birth weight is believed to reflect neonatal health in the perinatal period. The pathological considerations of the placenta provide biological evidence for the significance of birth weight [76]. Numerous studies have identified the risk factors for LBW. Therefore, LBW seems to be preventable. Moreover, birth weight may be associated with future morbidity, in addition to neurologic deficits and behavioral problems [77, 78].

### Health effects

This updated cohort profile provides evidence that exposure to environmental chemicals may be associated with not only fetus/infant but also maternal levels of hormones and FAs. Disruption of maternal hormone levels and FAs may consequently influence fetal and child health. Pregnant women are vulnerable to environmental exposure; thus, further investigation of their influence on maternal health is warranted. Regarding birth weight and birth size, we have added evidence of the lack of association between prenatal exposures to PCBs, Me-Hg, and DEHP, and birth weight. In addition to birth weight, the most common indicator of birth size, we also investigated the association of prenatal exposures with SGA, ponderal index values, and cord blood metabolic biomarkers such as adiponectin and leptin. Although no association was observed with birth weight, the risk of SGA by weight decreased with increased Me-Hg concentration [36]. However, no studies have suggested a reasonable assumption about the direct protective role of low Me-Hg exposure on in utero fetal growth. To our knowledge, the present study is the first to investigate the effect of prenatal DEHP exposure on both birth size and fetal metabolic biomarkers. The decreased ponderal index values observed in girls may be explained by decreased leptin levels.

Our findings on thyroid hormone levels imply that PFOS is more influential than PFOA on human thyroid hormones. The extended elimination time of PFOS could explain our finding. Our results indicate that prenatal exposure to PFASs and DEHP are associated with reproductive and steroid hormone levels. Our findings suggest that glucocorticoid and DEHA dyshomeostasis at birth are associated with in utero PFAS exposure, which may adversely affect the HPA axis and steroid hormone homeostasis in later life. Both PFOS and DEHP are known ligands of nuclear, estrogen, and androgen receptors, which are essential regulators of steroidogenesis [79–81]. The mechanism behind our observation could be explained by the aforementioned ligand properties of PFOS and DEHP.

We observed inverse and positive associations between bisphenol A and prolactin levels among boys and girls, respectively, yet studies on the association between bisphenol and prolactin are limited. Further studies are needed to identify the effect of prenatal exposure to bisphenol A on fetus reproductive hormones.

We found that prenatal PFOA exposure was negatively associated with mental development in girls at 6 months of age; however, no association was found between prenatal DEHP exposure and infant neurodevelopment at an early age. In our future work, postnatal environmental factors such as nutrition, daycare attendance, and access to educational resources should be taken into account, as these factors are known to influence child development. Continuous neurobehavioral development assessment throughout childhood will allow us to project the trajectory of child neurobehavioral development in our cohort studies. Inconsistent findings have been reported on the association between environmental chemical exposures and behavioral problems. The related symptoms at certain ages and evidence from prospective studies remain insufficient; thus, further studies are required.

The immunomodulatory [82] and immune-toxic [83, 84] effects of PFASs may impair immune function in humans, resulting in increased infectious disease prevalence and decreased allergic reactions. Future work should carefully investigate the trajectory of allergy and infectious diseases in association with prenatal exposure to environmental chemicals other than PFASs.

### Genetic susceptibility to environmental exposure

Our findings on gene polymorphisms suggest that the presence of maternal smoking [22, 61–63] and dioxin exposure [59] during pregnancy affects not only the genes encoding chemical metabolizing enzymes but also the genes encoding DNA repair possibly resulting in adverse fetal health effects, such as infant birth size. Our findings suggest that there are populations genetically susceptible to the effects of exposure to environmental chemicals. Policy on the regulation of environmental chemicals should be made in consideration of these susceptible populations.

### Epigenetic effects

We demonstrated that prenatal PFOA exposure resulted in reduced *IGF2* methylation in cord blood, which in turn was associated with reduced ponderal index values at birth [26], suggesting that exposure to environmental chemicals in utero may contribute to ponderal index via effects on DNA methylation. There is now compelling evidence that environmental factors may influence DNA methylation, suggesting that fetal exposure may contribute to lifelong health problems via these effects [12]. A number of epigenome-wide association studies (EWAS) have been conducted in humans to estimate the effect of maternal smoking. Recent meta-analyses of EWAS have reported that prenatal exposure to maternal smoking [85] and NO_2_ air pollution [86] influences offspring DNA methylation at birth. However, few studies have linked prenatal exposure to long-term response via DNA methylation.

### Future study directions

Firstly, investigations on the effects of multiple chemical exposures are warranted. Humans are constantly exposed to multiple environmental chemicals, and estimation of the combined risks of the mixture effects is required.

Secondly, the results of our study suggest that in utero exposure to environmental chemicals may contribute to infant health by affecting DNA methylation. To determine the potential long-term health effects of prenatal exposure via epigenetic mechanisms, a genome-wide array technology is valuable; however, few studies have used these approaches to assess the effects of prenatal exposure to environmental chemicals on child DNA methylation. We are conducting a genome-wide study to elucidate the association between prenatal chemical exposure and DNA methylation.

Thirdly, long-term follow-up of the participants is needed. The importance of the intrauterine and early childhood environment and later disease risk led to the establishment of the DOHaD theory. Children in the Hokkaido Study are now reaching the pubertal stage. The impact of fetal and early childhood exposures to environmental chemicals on neurobehavioral development, asthma and allergies, growth, and reproductive functions will be observed continuously and will require confirmation.

Lastly, the strengthening of the collaborations and integration with other birth cohort studies are important. The Birth Cohort Consortium of Asia (BiCCA) was launched in 2012, and 24 cohorts from 11 countries are currently participating [6]. Environmental risks or disease burdens vary from region to region. International cooperation among Asian cohort studies could help in exploring regional hazards, understanding the health impact of environmental toxicants, and developing effective prevention strategies. Although there are many challenges in facilitating different cohort studies, cooperative relationships will accelerate the establishment of new research within the BiCCA.

## Conclusions

The results of the Hokkaido Study suggest that even relatively low levels of exposure to environmental chemicals may have adverse effects on child health. When implementing policies and guidelines, it is important to consider individual genetic susceptibility to these effects. Further studies on the combined effects of chemical exposure, contribution to infant health via effects on DNA methylation, and long-term follow-up of the participants are essential.

## Abbreviations

ADHD-RS: Attention Deficit Hyperactivity Disorder-Rating Scale
AhR: Aromatic hydrocarbon receptor
ASQ: Autism Screening Questionnaire
ATS-DLD: American Thoracic Society-Division of Lung Disease
BiCCA: Birth Cohort Consortium of Asia
BMI: Body mass index
BSID-II: Bayley Scales of Infant Development second edition
CBCL: Child Behavior Checklist
CI: Confidence interval
Conners 3P: Conner’s 3rd Edition for Parents
CYP1A1: Cytochrome P450 1A1
CYP2E1: Cytochrome P450 2E1
DCDQ: Developmental Coordination Disorder Questionnaire
DDST: Denver Developmental Screening Tests
DEHP: di(2-Ethylhexyl) phthalate
DHA: Docosahexaenoic acid
DHEA: Dehydroepiandrosterone
DL: Detection limit
DL-PCB: Dioxin-like PCB
DOHaD: Developmental Origins of Health and Disease
EDCs: Endocrine-disrupting chemicals
EES: Evaluation of Environmental Stimulation
ELBW: Extremely low birth weight
EPA: Eicosapentaenoic acid
EWAS: Epigenome-wide association studies
FA: Fatty acid
FSH: Follicle-stimulating hormone
FT4: Free thyroxine
FTII: Fagan Test of Infant Intelligence
GSTM1: Glutathione *S*-transferase mu 1
IgA: Immunoglobulin A
IgE: Immunoglobulin E
IGF2: Insulin-like growth factor 2
INSL-3: Insulin-like factor 3
ISAAC: International Study of Asthma and Allergies in Childhood
J-PSAI: Japanese Pre-School Activities Inventory
K-ABC: Kaufman Assessment Battery for Children
KIDS: Kinder Infant Development Scale
LBW: Low birth weight
LH: Luteinizing hormone
LINE1: Long interspersed element 1
M-CHAT: Modified Checklist for Autism in Toddlers
Me-Hg: Methylmercury
MEHP: Mono(2-ethylhexyl) phthalate
MTHFR: Methylenetetrahydrofolate reductase
NQO1: Nicotinamide adenine dinucleotide phosphate quinone oxidase 1
OH-PCB: Hydroxylated PCB
ORs: Odds ratios
*p,p′*,: dichlorodiphenyldichloroethylene
PCB: Polychlorinated biphenyl
PCDD: Polychlorinated dibenzo-*p*-dioxin
PCDF: Polychlorinated dibenzofuran
PFASs: Perfluoroalkyl substances
PFDA: Perfluorodecanoic acid
PFDoDA: Perfluorododecanoic acid
PFHxS: Perfluorohexane sulfonate
PFOA: Perfluorooctanoic acid
PFOS: Perfluorooctanoic sulfonate
PFTrDA: Perfluorotridecanoic acid
SCQ: Social Communication Questionnaire
SDQ: Strengths and Difficulties Questionnaire
SES: Socioeconomic status
SGA: Small for gestational age
SHBG: Sex hormone-binding globulin
SNP: Single nucleotide polymorphism
TEQ: Toxicity equivalency quantity
TG: Triglyceride
TSH: Thyroid-stimulating hormone
VLBW: Very low birth weight
WAIS-R: Wechsler Adult Intelligence Scale-Revised
WCST-KFS: Wisconsin Card Sorting Test (Keio Version)
WISC-III: Wechsler Intelligence Scale for Children third edition
WISC-IV: Wechsler Intelligence Scale for Children fourth edition
XRCC1: X-ray cross-complementing gene 1
